# Preventive Effects of Evodiamine on Dexamethasone-Induced Osteoporosis in Zebrafish

**DOI:** 10.1155/2019/5859641

**Published:** 2019-01-22

**Authors:** Heng Yin, Jianwei Wang, Mao Wu, Yong Ma, Shanfu Wang, Qiuju Su

**Affiliations:** ^1^The TCM Hospital of Wuxi affiliated, Nanjing University of Chinese Medicine, Department of Traumatology & Orthopedics, China; ^2^Institute of Traumatology & Orthopedics, Nanjing University of Chinese Medicine, Nanjing, 210023, China; ^3^Nanjing Medical University, Affiliated Wuxi Peoples Hospital, Department of Acupuncture, China

## Abstract

The aim of this study was to investigate the effect of evodiamine (EV) on dexamethasone-induced osteoporosis in zebrafish. Zebrafish larvae were exposed to different concentrations of dexamethasone to obtain the osteoporosis in zebrafish. Calcium, phosphorus, and alizarin red staining determination were performed to evaluate the effects of EV on bone mineralization. Alkaline phosphatase (ALP), hydroxyproline (HP), and tartrate resistant acid phosphatase (TRAP) were also measured by commercial kits. The expression of MMP3-OPN-MAPK pathway in zebrafish was measured by Western blot. RT-PCR was used to determine mRNA levels of MMP3, OPN, and MAPK. EV could significantly increase the content of calcium and phosphorus. The results of alizarin red staining showed that EV could significantly increase the calcium sink of horse fish, increasing the area of bone formation. EV could increase the content of hydroxyproline in zebrafish. EV also increased ALP and TRAP in zebrafish. Western blot and RT-PCR results showed that EV restored the MMP3-OPN-MAPK pathway in zebrafish. In conclusion, we found that EV can alleviate dexamethasone-induced osteoporosis in zebrafish. The mechanism is related to activating MMP3-OPN-MAPK pathway and then activating bone remodeling.

## 1. Introduction

Osteoporosis is a systemic disorder of bone metabolism characterized by decreased bone density, increased bone fragility, and susceptibility to fracture. With the rapid development of economy, the aging of the population is becoming more and more serious, and the incidence of osteoporosis is also increasing year by year, which seriously threatens the health of the middle-aged and the elderly. The main complication of osteoporosis is fracture [1], which will seriously threaten the life and health of the middle-aged and elderly and will also bring heavy burden to daily life and social economy. The skeletons are in constant renewal, and this steady state of bone is mainly maintained by two kinds of cells, one is osteoclasts with bone resorption function and the other is osteoblasts with bone formation function. Osteoporosis can be caused if the bone resorption function of osteoclasts is stronger than that of osteoblasts. Therefore, the current treatment plan for osteoporosis is mainly aimed at resisting bone resorption or promoting bone formation [2]. However, there are few drugs that can resist bone resorption and promote bone formation at the same time, but they are expensive and have great side effects. The research of drugs with both functions has become a hot spot in this field.

Evodiamine is an alkaloid extracted from the fruit of Evodia rutaecarpa. Previous studies have shown that EV can alleviate the chronic high glucose challenge in inflammatory injury [3], protect septic mice from acute lung injury [4], inhibit tumor activity of several types of human cancers, and improve acute inflammatory cerebral ischemia [5]. However, the effect of EV on osteoporosis is rarely reported. Therefore, the purpose of this study was to evaluate the effect of EV on dexamethasone-induced osteoporosis in zebrafish.

## 2. Materials and Methods

### 2.1. Reagent

EV (purity: 97%) was purchased from the National Institute for the control of Pharmaceuticals and Biological Products (Beijing, china). Dexamethasone (Dex) and Alfacalcidol (AC) were purchased from Sigma-Aldrich (Darmstadt, Germany). Hydroxyproline (HP), alkaline phosphatase (ALP), and tartrate resistant acid phosphatase (TRAP) regent kits were supplied by R&D Systems (US). The primary antibodies were the products of Cell Signaling Technology (MA, USA).

### 2.2. Animal

The wild type AB is zebrafish, provided by Hangzhou Huante Biological Co., Ltd., of Zhejiang Province, and is bred in natural pairs. The age was 3 days after fertilization (3 DPF) with 180 pieces. Zebrafish was raised in 28°C water for fish culture (water quality: 200 mg of instant sea salt was added to every 1 L of reverse osmosis water, with conductivity of 480 ~ 510 *μ*s/cm; pH 6. 9-7. 2; the hardness 53. 7-71. 6 mg/L CaCO3).

### 2.3. Experimental Procedures

Zebrafish larvae were placed in a 24-well plate and randomly divided into 0.1% DMSO (control group), 10 *μ*mol/L Dex (model group), 10 *μ*mol/L Dex+ AC (0.1 *μ*g/kg), 10 *μ*mol/L Dex+ EV (50, 100 mg/kg) from 3 dpf to 9 dpf. At the end of the experiment, the scales and bone tissues of larvae were carefully collected under an anatomical stereomicroscope for subsequent examination.

### 2.4. Calcium and Phosphorus Detection

Zebrafish scales were cleaned and dried, dissolved with 70 % concentrated nitric acid, and then diluted 100 times with distilled water. The content of calcium and phosphorus in zebrafish was determined by inductively coupled plasma emission spectrophotometry. [6].

### 2.5. Determination of Hydroxyproline

Zebrafish scales were washed with distilled water and completely dried until they were constant in weight. Then, the sample was placed in a silica crucible for cracking and stably prepared at 800°C, and the weight of the cracked powder was recorded, hydrolyzed with 6 mol  /  L HCL, and finally detected with a hydroxyproline kit [6].

### 2.6. Determination of Alkaline Phosphatase Activity

Zebrafish scales were cut and treated with alkaline buffer containing 100 mM TRIS-HCl, pH 9.5, 1 mM magnesium chloride, and 0.1 mM zinc chloride for 30 minutes and then reacted with 150*μ*l alkaline buffer containing 20 mM 4-nitrobenzene disodium phosphate hexahydrate for 30 min. The reaction was terminated with a solution containing 3N NaOH and 20 mM EDTA and then measured by spectrophotometry at 405 nm [6].

### 2.7. Anti-Tartrate Acid Phosphatase (TRAP) Activity

Zebrafish scales were incubated with 0.1M sodium acetate buffer containing 20 mM tartrate for 1 hour and then further incubated with a solution containing 20 mM tartrate, 0.1M sodium acetate buffer, and 20 mM pNPP. The reaction was terminated with 50 *μ*L 2N NaOH. Finally, a standard curve was established with p-nitrophenol, the absorbance at 405 nm was measured, and the TRAP activity was indirectly reacted with the amount of p-nitrophenol (pNP) produced [6].

### 2.8. Alizarin Red Staining

After being cultured to 9dpf, zebrafish was fixed overnight with 4 % paraformaldehyde, the fish was bleached with 1% KOH bleach containing 1. 5% H_2_O_2_, and then alizarin red was used to stain the head bones of the zebrafish and then transparent with 1% KOH and glycerol with a gradient ratio of 1: 1 to remove excess stain. Finally, the photo was obtained digitally using a fluorescent stereomicroscope (40*∗*).

### 2.9. Quantitative Real-Time PCR Analysis

According to the manufacturer's instructions, the total RNA of the scales was extracted from the scales using the TRIZOL reagent (Invitrogen, Life Technologies, CA, USA). The purity of RNA was measured with 2000 thermal science nanodrops (Massachusetts, USA). Next, RNA was transcribed into cDNA using a reverse transcriptase kit (Takara Biotech, Kyoto, Japan). Quantitative real-time PCR (qRT-PCR) analysis was performed using a ChamQ SYBR qPCR Master Mix (Vazyme Biotech Co., Ltd., Nanjing, China) with the CFX Manager software (Bio-Rad Laboratories Inc.). GAPDH was analyzed in each sample for standardized expression. The primers used in this study are listed in Table 1. The relative expression was analyzed by 2 ^−ΔΔCt^ .

### 2.10. Determination of MMP3-OPN-MAPK Pathway Related Proteins

The total protein was extracted from zebrafish larvae by RIPA lysate, centrifuged at 12,000 rpm for 15 min, and the supernatant was collected. The BCA kit was used to quantify each group of proteins. Each protein sample was subjected to SDS-polyacrylamide gel electrophoresis and then transferred to PVDF membrane. The PVDF membrane was then sealed in 5 % skim milk for 2 h. After 2 hours of blocking, the PVDF membrane was placed in the corresponding primary antibody and incubated at 4°C overnight. The next day TBST was washed 4 times for 8 min each time. The PVDF membrane was then placed in a secondary antibody solution and incubated for 2 h at room temperature in a shaker. The PVDF membrane was removed and washed 4 times with TBST for 8 min each time. The gray value of each strip was analyzed by exposing and scanning the strip using a gel imaging system.

### 2.11. Statistical Analysis

Statistical analysis was carried out using SPSS 16.0 software, and the data were expressed in mean values ± SDs, using one-way ANOVA analysis. In two-to-two comparisons between groups By LSD method, p<0.05 was statistically different.

## 3. Results

### 3.1. Effect of EV on Calcium and Phosphorus

**Figure 1:**
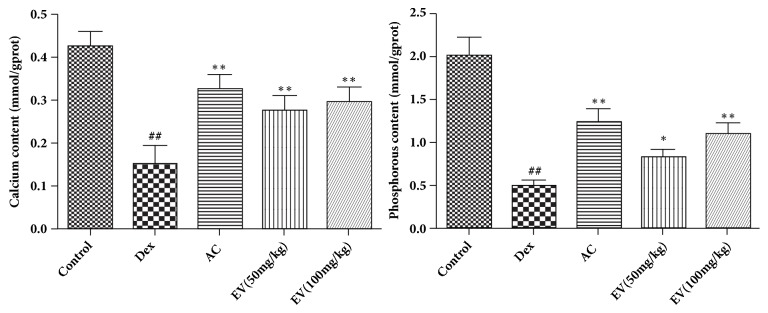
**Effect of EV on calcium and phosphorus.** All values are expressed as means ± SDs. ^#^P<0.05, ^##^P<0.01 vs. control group. *∗*P<0.05,*∗∗*P<0.01 vs. model group.

As shown in Figure 1, Dex significantly reduced calcium and phosphorus in contrast with normal zebrafish. On the contrary, EV and AC significantly increased calcium and phosphorus in zebrafish.

### 3.2. Effect of EV on Hydroxyproline

**Figure 2:**
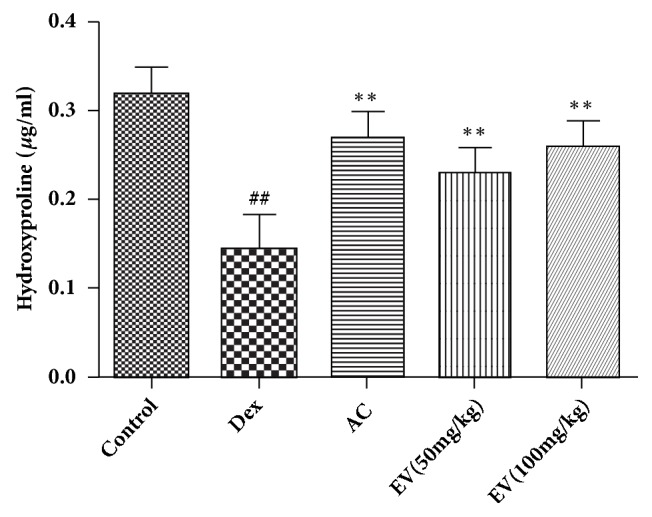
**Effect of EV on hydroxyproline.** All values are expressed as means ± SDs. ^#^P<0.05, ^##^P<0.01 vs. control group. *∗*P<0.05,*∗∗*P<0.01 vs. model group.

As shown in Figure 2, Dex significantly downregulated hydroxyproline in contrast with normal zebrafish. On the contrary, EV and AC significantly increased hydroxyproline in zebrafish.

### 3.3. Effect of EV on ALP and TRAP Activities

**Figure 3:**
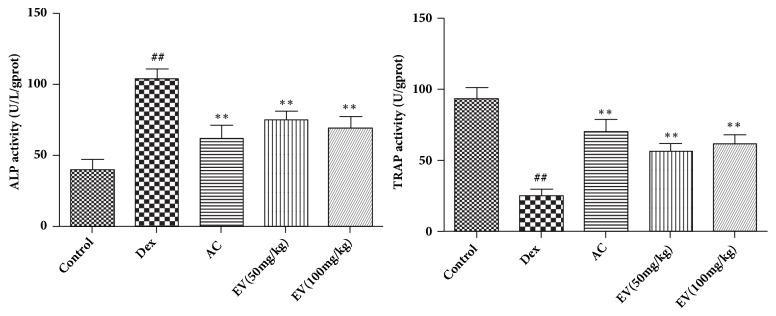
**Effect of EV on ALP and TRAP activities.** All values are expressed as means ± SDs. ^#^P<0.05, ^##^P<0.01 vs. control group. *∗*P<0.05,*∗∗*P<0.01 vs. model group.

As shown in Figure 3, Dex significantly decreased ALP and increased TRAP in contrast with normal zebrafish. On the contrary, EV and AC significantly increased ALP and decreased TRAP in zebrafish.

### 3.4. Effect of EV on Alizarin Red Staining

**Figure 4:**
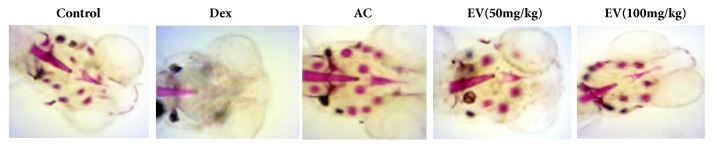
**Effect of EV on alizarin red staining.** All values are expressed as means ± SDs. ^#^P<0.05, ^##^P<0.01 vs. control group. *∗*P<0.05,*∗∗*P<0.01 vs. model group.

Figure 4 showed that, compared with the control group, the alizarin red staining area of zebrafish skulls in the model group gradually decreased, and the area and cumulative optical density of zebrafish skulls in the EV and AC groups were significantly increased compared with the model group.

### 3.5. Effect of EV on MMP3, OPN, and MAPK mRNA Levels

**Figure 5:**
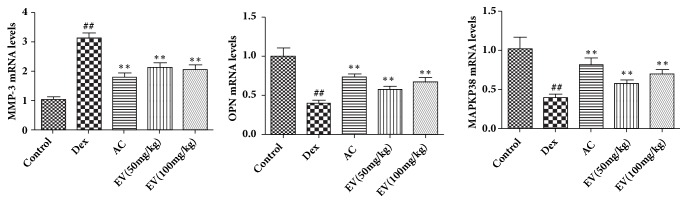
**Effect of EV on MMP3, OPN, and MAPK mRNA levels.** All values are expressed as means ± SDs. ^#^P<0.05, ^##^P<0.01 vs. control group. *∗*P<0.05,*∗∗*P<0.01 vs. model group.

As shown in Figure 5, Dex significantly decreased the mRNA levels of OPN and MAPKp38 and increased MMP3 compared with control group. On the contrary, EV and AC significantly increased the mRNA levels ofOPN and MAPKp38 and decreased MMP3 in zebrafish.

### 3.6. Effect of EV on the Protein Expressions of MMP3, OPN, and MAPK

**Figure 6:**
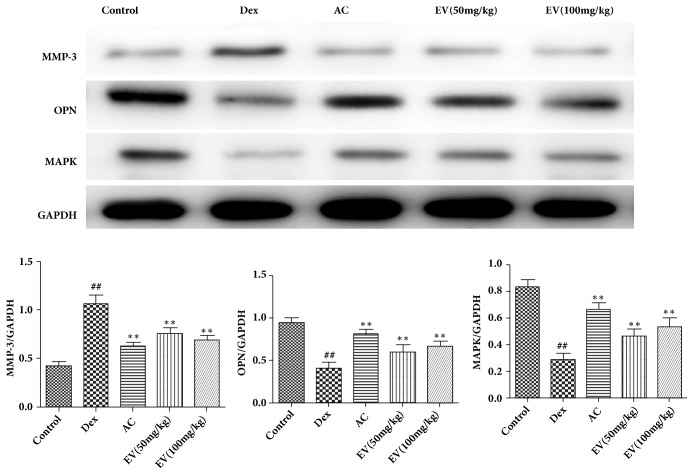
**Effect of EV on the protein expressions of MMP3, OPN, and MAPK.** All values are expressed as means ± SDs. ^#^P<0.05, ^##^P<0.01 vs. control group. *∗*P<0.05,*∗∗*P<0.01 vs. model group.

As shown in Figure 6, Dex significantly decreased the protein expressions of OPN and MAPKp38 and increased MMP3 compared with control group. On the contrary, EV and AC significantly increased the protein expressions of OPN and MAPKp38 and decreased MMP3 in zebrafish.

## 4. Discussion

Osteoporosis is a systemic bone disease characterized by decreased bone mass and destruction of bone tissue microstructure, characterized by increased bone fragility and susceptibility to fracture [7]. With the aging of our population and the increasing incidence of osteoporosis, it has seriously threatened human health [8]. In this study, dexamethasone was used to establish a zebrafish osteoporosis model to evaluate the pharmacological and mechanism of EV against osteoporosis.

The replacement of bone must be carried out continuously to ensure its strength and integrity. At the same time, two conflicting activities will affect bone remodeling. These are osteoblasts to produce organic bone matrix and osteoclasts to dissolve bone minerals and matrix [9]. Maintaining the balance between bone formation (osteoblasts) and bone resorption (osteoclasts) is the main factor leading to bone metabolism. Once bone resorption exceeds the stratum, the risk of osteoporosis and fracture in bone increases [10]. In the current study, the activity of TRAP in scales was detected to evaluate the activity of osteoclasts. TRAP is a marker of osteoclast activity (bone resorption) and can be hydrolyzed in the presence of resorption pits. ALP activity is usually used to measure osteoblast differentiation. Biochemical tests of TRAP and ALP activity showed that Dex significantly decreased ALT and increased TRAP in contrast with normal zebrafish. On the contrary, EV and AC significantly increased ALT and decreased TRAP in zebrafish, which means that the recovery of bone formation/bone resorption homeostasis is closer to the level of control group.

The regulation of matrix metalloproteinases (MMPs) on osteoclast bone resorption has attracted more and more attention [11]. MMPs are a group of proteolytic enzymes that depend on Zn^2+^ hydrolysis. Up to now, it has been found that MMP family only degrades fibrillar collagen. As three members of the MMP family, MMP3, MMP9, and MMP13, play an important role in the activation of osteoclasts [12]. It is reported that MMP3 degrades type I collagen barrier bone cells, followed by active osteoclasts [13]. In this study, Dex significantly increased the mRNA and protein level of MMP3, compared with control group. On the contrary, EV and AC significantly decreased the mRNA and protein level of MMP3 in zebrafish.

Osteoblasts can secrete OPN and play an important role in the mineralization of bone matrix. There is an aspartic acid-rich region in OPN molecule, through which OPN can bind to light apatite in tissue and play a role [14]. After the bone matrix mineralization began, the OPN level in osteoblasts began to increase, and the bone line and periosteum plate of OPN had a higher concentration in the process of bone reconstruction, which means that OPN plays an important role in the process of osteoblast adhesion and mineralization termination [15]. Therefore, OPN signaling pathway is an important medium for regulating osteoblast behavior. In this study, Dex significantly decreased the mRNA and protein level of OPN, compared with control group. On the contrary, EV and AC significantly increased the mRNA and protein level of OPN in zebrafish.

The proliferation, differentiation, and apoptosis of bone cells in bone metabolism all depend on transmembrane signaling, thus linking cell signaling with bone metabolism. Mitogen-activated protein kinase (mitogen-activated protein kinase, MAPK) signal pathway is an important pathway for extracellular signal to induce nuclear reaction [16]. It consists of a cascade of activated silk/threonine protein kinases. In the process of signal transmission, the phosphorylation of amino acid residues is activated step by step [17]. Recent studies have found that MAPK is closely related to the proliferation, differentiation, and apoptosis of osteoblasts [18]. Recent studies have found that the decreased expression of MMP3 protein can further activate OPN expression on the cell membrane, promote the conduction of MAPK in the cell, and enhance the activity of osteoblast cell. At the same time, studies at home and abroad show that extracellular MMP3 of osteoblasts can activate OPN protein as a cleavage protein, thus promoting downstream signal protein, activating signal transduction pathway, and enhancing cell activity and proliferation through MAPK [19]. In this study, Dex significantly decreased the mRNA and protein level of MAPK, compared with control group. On the contrary, EV and AC significantly increased the mRNA and protein level of MAPK in zebrafish.

To sum up, we demonstrated that EV may prevent osteoporosis by reversing the imbalance of bone formation/bone resorption and activating MMP3-OPN-MAPK pathway signal. Further study was necessary for the therapy of osteoporosis with EV.

## Figures and Tables

**Table 1:** Primers used in this study.

Genes	Primer sequence (5′-3′)	Primer sequence (3′-5′)
MMP3	CTCCCCACCTTGAATGAAGA	ACTGGGTCGCTTCTCTTGAA
OPN	AACCCAGACACAAGCATTCC	GCCTTTGAGGTTTTTGGTCA
MAPK	ATTGTCAGCAATGCATCCTG	ATGGACTGTGGTCATGAGCC
GAPDH	AGCAATGCTTGTCAATCCTG	ATTGGTCGGACTGATGAGCC

## Data Availability

The data used to support the findings of this study are available from the corresponding author upon request.
